# The Impact of Submicroscopic Parasitemia on Malaria Rapid Diagnosis in Northeastern Tanzania, an Area with Diverse Transmission Patterns

**DOI:** 10.3390/idr14060082

**Published:** 2022-10-25

**Authors:** Robert Diotrephes Kaaya, Johnson Matowo, Debora Kajeguka, Filemoni Tenu, Boniface Shirima, Franklin Mosha, Reginald Kavishe

**Affiliations:** 1 Department of Medical Parasitology and Entomology, Kilimanjaro Christian Medical University College, Moshi P.O. Box 2240, Tanzania; 2 Pan-African Malaria Vector Research Consortium, Moshi P.O. Box 2240, Tanzania; 3 Department of Biochemistry and Molecular Biology, Kilimanjaro Christian Medical University College, Moshi P.O. Box 2240, Tanzania

**Keywords:** malaria transmission, sub-microscopic, low-density, parasitemia, rapid diagnostic test

## Abstract

Global malaria epidemiology has changed in the last decade with a substantial increase in cases and deaths being recorded. Tanzania accounts for about 4% of all cases and deaths reported in recent years. Several factors contribute to the resurgence of malaria, parasite resistance to antimalarials and mosquito resistance to insecticides being at the top of the list. The presence of sub-microscopic infections poses a significant challenge to malaria rapid diagnostic tests (mRDT). Our cross-sectional surveys in Handeni and Moshi, Tanzania assessed the effect of low parasite density on mRDT. Handeni had higher malaria prevalence by mRDT (39.6%), light microscopy (LM) (16.9%) and polymerase chain reaction (PCR) (18.5%), compared to Moshi with prevalence of 0.2%, 1.3% and 2.3%, respectively. A significant difference (*p* ˂ 0.001) in malaria prevalence by mRDT, LM and nested PCR was found among age groups. In comparison to all other groups, school-age children (5–15 years) had the highest prevalence of malaria. Our results show that mRDT may miss up to 6% of cases of malaria mainly due to low-density parasitemia when compared to LM and PCR. Routinely used mRDT will likely miss the sub-microscopic parasitemia which will ultimately contribute to the spread of malaria and hinder efforts of elimination.

## 1. Introduction

In recent years, there has been little progress in the fight against malaria globally and malaria cases and deaths are on the rise [1]. According to the World malaria report of 2021, malaria cases and deaths increased by about 14 million and 67,000 respectively from 2019 to 2020 [1]. The World Health Organization (WHO)-African region contributes to 95% of global cases, with six sub-Saharan countries accounting for about 55% of the cases globally [1]. National malaria surveillance systems in sub-Saharan Africa (SSA) countries are centered on the detection of symptomatic cases by either mRDTs or LM (LM) [2]. Symptomatic cases fluctuate during transmission season and usually the peak is just after the rainy season which correlates well with an increase in *Anopheles* mosquito densities [3,4]. Studies suggest that a history of previous malaria exposure can protect some people from the severe manifestation of the disease particularly in high transmission areas [5]. There is evidence that naturally-acquired immunity against malaria parasites can inhibit merozoite invasion of the red blood cells (RBCs) and thus suppress the multiplication of the parasites to maintain low-density parasitemia [6,7].

Individuals with asymptomatic and sub-microscopic infections rarely seek medical treatment and they can act as potential reservoirs for malaria transmission [8,9,10,11]. Sub-microscopic infections have been suspected to facilitate human–mosquito transmission in low endemic areas [12]. Studies suggest that the right gametocyte micro to the macro ratio of fewer than 5 gametocytes/µL is enough for transmission to occur [13,14]. This sexual parasite density is below the detection threshold of microscopic analysis.

The low-density parasitemia will likely contribute to the new transmission circles when mosquito numbers increase in the rainy seasons [9]. Sub-microscopic infections pose a serious challenge to parasite detection as a prerequisite for malaria treatment.

The introduction of mRDT in Tanzania more than ten years ago led to the replacement of LM in most health facilities [15]. Although mRDT has more advantages over LM, it still faces some operational challenges throughout its supply chain [16]. WHO recommends procurement of mRDTs with at least a 75% panel detection score (PDS) at 200 parasites/µL. It is very likely that, with the WHO-recommended mRDT limit of detection, 25% of infections will be missed and sustain ongoing transmission [10,17]. Microscopy has a detection limit of 50 parasites/µL, which is better than mRDT, while nucleic acid detection methods such as PCR are the most sensitive with a detection threshold of approximately 0.2 parasites/µL [18,19].

This study aimed at investigating the impact of sub-microscopic malaria infections on mRDT in comparison with LM and PCR across areas with diverse malaria transmission endemicity.

## 2. Materials and Methods

### 2.1. Description of the Study Areas

**Figure 1:**
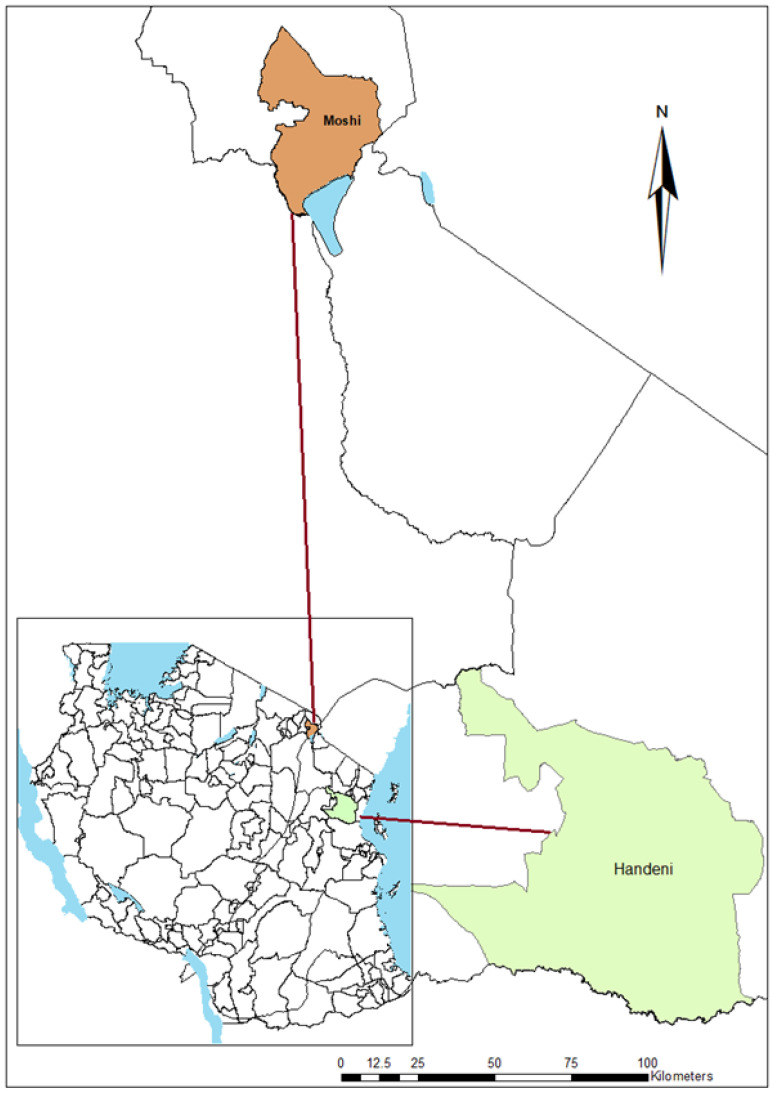
A map of Tanzania showing the study sites (map created using ArcGIS software v10.3).

The study was conducted in Handeni, Tanga region and Moshi in Kilimanjaro region (Figure 1). Handeni is endemic to malaria with a perennial transmission pattern and prevalence of about 28% [20]. The study area has two rainy seasons per year which denotes the peaks of malaria transmission. The long rainy season is from March–June and the short rainy season is from October–November. The area is located 309 m above sea level. Residents in Handeni engage themselves in small-scale farming and livestock husbandry.

The second study site, Moshi, is located 10 km from Moshi municipality, and 800 m above sea level, south of Mount Kilimanjaro. Most of the population in the area is engaged in agriculture with irrigated rice and sugarcane cultivation as the main crops. Irrigation activities provide an important breeding site for *Anopheles arabiensis*, which are the secondary vectors in malaria transmission. Lower Moshi is a low malaria-endemic area with a prevalence of about 1% in the last 10 years [21].

Participants of the study were residents from the two areas with an age of 6 months and above.

### 2.2. Participants Recruitment and Sample Collection

A community sensitization campaign was carried out in the study areas, where village residents were invited to designated dispensaries to participate in the study. Potential participants were informed in detail of the study and invited to voluntarily participate in the study by signing an informed consent form. About 1003 participants gave consent to participate, and their demographic information were collected following a questionnaire.

A finger prick blood was collected for mRDT, microscopy and dried blood spots. Blood spots were prepared from 50 µL of blood on Whatman^®^ protein saver cards (Cytiva Plc, Marlborough, MA, USA) followed by drying at room temperature overnight. Dried filter papers were stored in a bag containing silica gel at room temperature.

Sampling in both the study sites was done at the end of the long rainy season between April and June 2018.

### 2.3. Malaria Rapid Diagnosis (mRDT)

Malaria detection on site was done using SD BIOLINE pf/pan Ag test kit (Standard Diagnostic INC-Korea) following testing procedures as per manufacturer’s instructions. Participants found to be positive were treated using Artemether-lumefantrine (ALu), the recommended first-line antimalarial treatment for uncomplicated malaria in Tanzania.

### 2.4. Light Microscopy

Thick and thin blood smears were prepared using freshly collected blood. The thin smear was fixed using absolute methanol. Both smears were stained for 10 min with 10% Giemsa solution, washed with distilled water and dried on a slide warmer (Fischer Scientific, Waltham, MA, USA). Slides were packed in slide boxes and stored at room temperature and the reading took place within 7 days after preparation. Slide reading and recording were done by qualified microscopists who are examined for competence 3 times a year with control slides from College of American Pathologists (CAP). Parasite/µL was estimated by the function of parasite counted against 200 white blood cells in thick smear and 8000 divided by the number of leucocytes as described in the WHO protocols [22]. Parasitemia classification was as follows; Low parasitaemi < 1000 parasites/µL, moderate parasitaemi 1000–4999 parasites/µL and High parasitemia > 5000 parasite/µL.

### 2.5. Polymerase Chain Reaction (PCR)

DNA for the Polymerase Chain Reaction was extracted following a modified-chelex method [23], where filter paper discs of 8 mm diameter were cut from the dried blood spots and placed in a 1.5 mL microcentrifuge tube and 1 mL of 0.5% Saponin/Phosphate Buffered Saline (PBS) solution was added to each sample tube, inverted several times and incubated at 4 °C overnight to remove haem from the filter paper discs. On the following day, all 0.5% saponin/PBS solution was aspirated and 1.0 mL cold PBS solution was added to the filter paper containing tubes followed by vortexing for 15 s and incubation at 4 °C for 15 min after which the PBS solution was aspirated and discarded. 150 µL of 6% chelex solution in DNase/RNase-free water was added to each sample. Samples were then heated in a heat block at 100 °C for 30 min followed by spinning at 12,000 rpm for 5 min to settle down chelex. About 100 μL of the supernatant (DNA-containing solution) was transferred into a new 1.5 mL microcentrifuge tube and stored at –20 °C until use.

*Plasmodium* species were detected using a nested-PCR (PCR) technique targeting 18S ribosomal RNA, specifically for *P. falciparum, P. malariae, P. vivax and P. ovale* identification as previously described by Snounou et al. [24].

### 2.6. Statistical Analysis

Statistical analysis was conducted using Stata 16 software (Stata Corp LLC, Dallas, TX, USA).

Parasitemia was log-transformed and presented in a scatter plot. Proportions and frequencies were presented in tables and figures. Wilcoxon rank-sum (Mann-Whitney) test was used to test statistically significant differences between age groups if *p* < 0.05. A two-sample test of proportions (prtest) was used to determine if there were statistically significant differences in prevalence (proportions) between groups

Cohen’s kappa statistic was used to measure reliability of PCR, RDT and LM results with 95% confidence intervals. Positive likelihood and negative likelihood ratio tests were calculated as follows [25]:

$$\mathrm{Positive}\;\mathrm{likelihood}\;\mathrm{ratio}\;\mathrm{test}=\frac{\mathrm{SENSITIVITY}}{\left(1-\;\mathrm{SPECIFICITY}\right)}$$

$$\mathrm{Negative}\;\mathrm{likelihood}\;\mathrm{ratio}\;\mathrm{test}=\frac{\mathrm{SPECIFICITY}}{\left(1-\;\mathrm{SENSITIVITY}\right)}$$

Total agreement percent was calculated as the sum of true positives and true negatives divided by total tests. 

## 3. Results

### 3.1. Malaria Prevalence by mRDT, LM, and PCR

The study enrolled 1003 participants, but we only included 985 participants in this analysis, who had complete data collected, of which 513 (52.1%) were from Handeni and 472 (47.9%) from Moshi. The majority of the participants were over 15 years of age, constituting 54.1%. Children aged 5–15 years and ˂5 years comprised 23.5% and 22.4%, respectively. 

Parasite prevalence by mRDT, LM and PCR was significantly higher in Handeni (*p* ˂ 0.001) when compared to Moshi. Furthermore, when comparing the individual tests within sites, mRDT detected a significantly higher proportion of malaria cases in the Handeni site than LM and PCR (*p* ˂ 0.001) different from the Moshi site, where PCR detected more malaria cases than RDT and LM (*p* ˂ 0.001). There were also significant differences in malaria prevalence across age groups in Handeni. School-age children (5–15 years) had a higher proportion of malaria cases than the rest of the age groups (*p* ˂ 0.001) in the Handeni site. Malaria prevalence in Moshi was too low to perform an age-wise analysis **(**Table 1).

### 3.2. Parasitemia Levels across Age Groups

**Figure 2:**
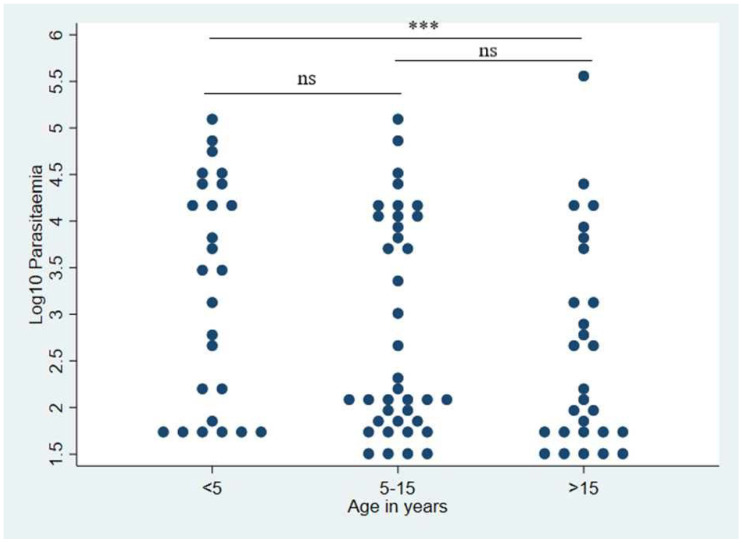
A comparison of parasitemia across age groups in both Handeni and Moshi. Significance differences are shown as: *** (Significant, *p*-value = 0.0284), ns (not significant) using Wilcoxon rank-sum test and n = 92.

Children under five years of age had the highest median parasitemia (3162 p/µL) when compared to other age groups. There was a significant difference in parasitemia between participants aged below 5 years and those aged >15 years (*p*-value = 0.0284), with the lowest parasitemia of 159 p/µL and 100 p/µL, respectively. There was no significant difference in parasitemia between school-age children and those below five years of age (Figure 2).

### 3.3. Sub-Microscopic Parasitemia

**Figure 3:**
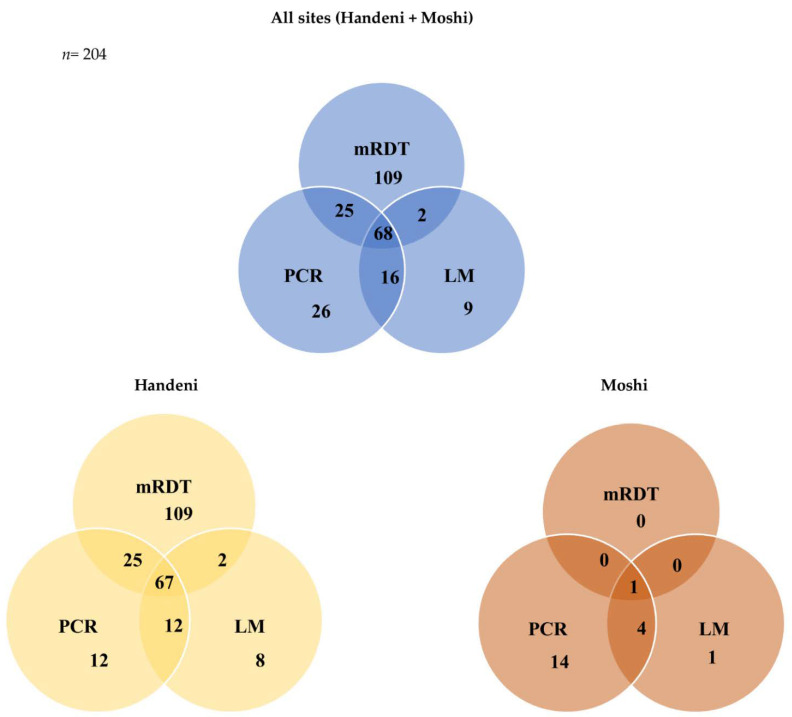
A Venn diagram showing overlaps in malaria detection by different diagnostic tools in the study sites.

Submicroscopic parasitemia refers to the samples negative by LM and mRDT (assuming detection thresholds are the same) but positive by PCR. Of the 204 total positive samples in all study sites, (26) 13% were positive by PCR alone, and almost equally distributed in Handeni and Moshi (12) 46% and (14) 54%, respectively. Most of the samples had low parasitemia, 22 of the 26 (85%) had parasite density below 200 p/µL and only two samples had high parasitemia. More than 58% (15) of sub-microscopic cases were from participants 15 years and above followed by school-age children (39%) and children below 5 years of age (3%) (Figure 3).

### 3.4. Diagnostic Accuracy of the LM, mRDT and PCR

The performance of the mRDT to detect malaria was different when compared to LM and PCR in Handeni and Moshi sites. The sensitivity and specificity of mRDT were 75.3% and 85%, respectively, when compared to PCR results. Compared to PCR, LM had a slightly higher sensitivity and specificity than mRDT. The positive predictive value for mRDT (34.31%) was almost half that of LM when compared to PCR (62.22%). RDT and LM showed similar negative predictive values. LM had a higher relative agreement value of around 94% with PCR at κ of 0.7 (Table 2).

## 4. Discussion

In this community-based study, we report a considerable difference in malaria prevalence between the study sites. Handeni had a significantly higher prevalence when compared to Moshi, and generally there were no statistically significant difference in malaria prevalence between female and male participants. Based on the historical malaria transmission patterns in Handeni, a lowland inhabited by anthropophilic *Anopheles gambiae ss* and *Anopheles funestus* mosquitoes [26,27], *Anopheles arabiensis*, which are zoophilic in nature, are abundant in Moshi, with a low transmission rate [28]. These results are consistent with recent studies that were conducted in the study sites which reported almost similar findings [20,21,29].

Rapid test (mRDT) detected more cases in Handeni compared to LM and PCR, suggesting the presence of mRDT false positivity. This was different from the Moshi site, where PCR detected more cases than mRDT suggesting the presence of submicroscopic parasitemia. Observation from several studies in high malaria transmission areas estimates up to 5% false positivity due to circulating HRP2 antigen up to five weeks after malaria treatment [30]; this could explain the results we observed in Handeni which is a high to moderate transmission area [20]. Some studies have also highlighted mRDT cross-reactivity with immunological agents such as rheumatoid factor (RF) and Human-Anti-Mouse Antibody (HAMA), whereby a 20.7% false positivity was reported [31,32]; ideally, this might falsely inflate the sensitivity score of mRDT.

False positivity ramifications are far less compared to false negativity. False-positive individuals will still receive malaria treatment instead of for the actual disease they are suffering from. Treating false-positive malaria cases wastes resources and delays the treatment of non-malaria conditions [31].

As compared to PCR, which is a more sensitive diagnostic tool and a comparator in this analysis, mRDT is relatively less sensitive, although low agreement was observed when compared to LM and PCR. In this study, mRDT gave positive results to 75 out of every 100 true positive individuals who lived within the study area. The low sensitivity of mRDT may be due to submicroscopic parasitemia, which cannot be detected by mRDT [33,34,35]. According to other studies, SD Bioline Pf/PAN antigen tests showed relatively high sensitivities of >95% [36,37].

An age-dependent prevalence analysis showed that school-age children had higher malaria prevalence than the other groups, even though they represented only a quarter of all study participants. Children under the age of five and pregnant women are the main targets of malaria interventions. There is a significant disparity in the delivery of bed nets whereby school-age children are prioritized the least, despite being equally at risk, and this could be the reason for their high malaria prevalence [38,39]. This analysis was not carried out in Moshi due to a very low malaria prevalence. Several studies have noted an increase in the risk of malaria infection among school-age children over the past decade, and recommendations are made to include this age group too as vulnerable [38,40,41].

Findings from this study showed a high proportion of sub-microscopic parasitemia in adults (>15 years) and children of age between 5 and 15 years. This explains the possibility that adults and children of age 5 to 15 years might be experiencing repeated malaria exposure and can sustain sub-microscopic parasitemia [42]. Asymptomatic sub-microscopic parasitemia was more prominent in adults but recently we have observed a paradigm shift to school-age children, making them a potential parasite reservoir [43,44].

A sub-microscopic infection results from immunological inhibition of parasite multiplication [45,46,47,48]. As a result of exposure to parasites over a long period of time, it is likely that adults would have protective immunity to severe manifestations of the disease [49,50]. Observations from studies in SSA suggest that more frequent exposure to malaria in school-age children may have led to increased malaria parasite-specific immunity and asymptomatic parasite persistence [51,52].

Results from this study showed that mRDT failed to detect six cases and four cases in every 100 participants in Handeni and Moshi, respectively, when compared to LM and PCR combined. Since we have already submitted data on *pfhrp2* deletion, we ruled out the possibility of false negatives due to *pfhrp2* deletion. The high detection threshold of mRDT compared to LM and PCR could also support this finding [53]. This raise concerns as to whether mRDT is the appropriate diagnostic tool in low malaria-endemic areas like Moshi. Findings elsewhere in SSA have demonstrated the possibility of mRDT missing malaria cases due to the low limit acceptable detection threshold set by WHO, and this could have ramifications in elimination campaigns [54,55,56].

This cross-sectional study reported a substantial proportion of cases of low parasitemia (˂200 p/µL) from Handeni and Moshi. There were also two cases with high parasitemia (>300,000 p/µL) and negative mRDT results. There have been studies showing that these submicroscopic cases tend to be asymptomatic [57,58]. It is thought that high parasitemia will produce a false negative result in mRDT because of the prozone effect [59,60].

False-negative mRDT is common in low-endemic areas and is believed to have implications for malaria elimination. The evidence of sub-microscopic infections in low malaria-endemic areas suggests that up to 50% of seasonal transmission is attributed to these infections [43]. Studies that aimed at mass testing and treatment failed to show the reduction of malaria transmission due to the use of mRDT as a diagnostic tool in areas earmarked for pre-elimination [61,62]. Clusters of sub-microscopic infections are stable through low and high transmission seasons. Targeting these clusters can significantly reduce malaria transmission and warrant success in malaria control and elimination.

### Study Limitations

RDT performance was compared only for LM and PCR since these are the most commonly used diagnosis methodologies for malaria detection, especially in research. We did not test other techniques such as Loop-mediated Isothermal Amplification (LAMP) which is a nucleic acid amplification assay, which is sensitive, rapid and cheaper compared to PCR. In this study we did not test for immunological factors that might give mRDT false-positive results.

## 5. Conclusions

There is evidence that sub-microscopic parasitemia infections occur in both high and low malaria transmission settings. Although mRDT is easy to use and convenient in low resource settings, it has a higher detection threshold than LM and PCR, making it less viable in low-endemic settings with high sub-microscopic malaria prevalence. The PCR technique is sophisticated and expensive to operate making it inappropriate for large scale deployment in low income countries. Although LM is usually referred to as a gold standard for malaria diagnosis, lack of expertise has been the greatest challenge. Sub-microscopic malaria will ultimately facilitate human-mosquito transmission and the effect is more pronounced in low-endemic areas which hinders elimination campaigns. A comparative evaluation of the relative benefits of mRDT should be conducted in areas where malaria prevalence is less than 5%.

## Figures and Tables

**Table 1:** Malaria prevalence by mRDT, LM and PCR across age groups in Handeni and Moshi sites.

mRDT					LM				PCR			
*n =* 998	Prevalence n (%)	95% C.I.		*p*-Value	Prevalence n (%)	95% C.I.		*p*-Value	Prevalence n (%)	95% C.I.		*p*-Value
		Lower	Upper			Lower	Upper			Lower	Upper	
**Sex**												
Male	54 (19.9)	15.1	24.6	0.723	21 (7.7)	4.5	10.9	0.298	35 (12.9)	8.9	16.8	0.7816
Female	148 (20.9)	17.9	23.9		70(9.9)	7.7	12.1		96 (13.5)	11.0	16.1	
**Age in years**												
<5	55 (25.3)	19.6	31.1	<0.001	26 (12.0)	7.7	16.3	0.001 *	34 (15.7)	10.8	20.5	<0.001 *
5–15	97 (42.5)	36.1	49.0		39 (17.1)	12.2	22.0		58 (25.4)	19.8	31.1	
>15	51 (9.7)	7.2	12.3		28 (5.3)	3.4	7.3		40 (7.6)	5.4	9.9	
**Study site (*n*)**												
Handeni (513)	203 (39.6)	35.3	43.8	<0.001	89 (16.9)	13.7	20.1	<0.001	116 (22.1)	18.5	25.6	<0.001
Moshi (472)	1 (0.2)	−0.2	0.6		6 (1.3)	0.3	2.3		19 (4.0)	2.3	5.8	

* Although the overall prevalence of malaria across all age groups was significant using the prtest, the confidence intervals for age < 5 and 5–15 overlap, showing no statistical difference for these two age groups.

**Table 2:** Performance comparison between mRDT, LM and PCR in the study sites.

	Sensitivity Value 95% C.I.	Specificity Value 95% C.I.	PPV Value 95% C.I.	NPV Value 95% C.I.	Likelihood ratio		Agreement (%)	Kappa Value 95% C.I.
					Positive Test Value 95% C.I.	Negative Test Value 95% C.I.		
PCR vs. LM	88.42	94.35	62.22	98.73	15.65	8.15	93.79	0.70
	86.44–90.41	92.92–95.78	59.21–65.23	98.03–99.42	12.21–21.42	6.85–9.99		0.63–0.77
PCR vs. mRDT	75.27	84.98	34.31	97.06	5.01	3.44	84.67	0.39
	72.57–77.96	82.75–87.21	31.35–37.28	96.00–98.11	4.21–6.10	3.02–3.96		0.32–0.47
LM vs. mRDT	34.31	97.06	75.27	84.98	11.67	1.48	84.06	0.39
	31.35–37.28	96.00–98.11	72.57–77.96	82.75–87.21	7.84–19.72	1.40–1.56		0.32–0.47

## Data Availability

Data sets developed during this investigation are not publicly available, but can be requested from the corresponding author upon reasonable notice.

## References

[1] World Health Organization (2021). World Malaria Report 2021.

[2] World Health Organization (2018). Malaria Surveillance, Monitoring and Evaluation: A Reference Manual.

[3] Ouédraogo A.L., de Vlas S.J., Nébié I., Ilboudo-Sanogo E., Bousema J.T., Ouattara A.S., Verhave J.P., Cuzin-Ouattara N., Sauerwein R.W. (2008). Seasonal Patterns of Plasmodium Falciparum Gametocyte Prevalence and Density in a Rural Population of Burkina Faso. Acta Trop..

[4] Geiger C., Agustar H.K., Compaoré G., Coulibaly B., Sié A., Becher H., Lanzer M., Jänisch T. (2013). Declining Malaria Parasite Prevalence and Trends of Asymptomatic Parasitaemia in a Seasonal Transmission Setting in North-Western Burkina Faso between 2000 and 2009–2012. Malar. J..

[5] Dent A.E., Nakajima R., Liang L., Baum E., Moormann A.M., Sumba P.O., Vulule J., Babineau D., Randall A., Davies D.H. (2015). Plasmodium Falciparum Protein Microarray Antibody Profiles Correlate with Protection from Symptomatic Malaria in Kenya. J. Infect. Dis..

[6] Niang M., Bei A.K., Madnani K.G., Pelly S., Dankwa S., Kanjee U., Gunalan K., Amaladoss A., Yeo K.P., Bob N.S. (2014). STEVOR Is a Plasmodium Falciparum Erythrocyte Binding Protein That Mediates Merozoite Invasion and Rosetting. Cell Host Microbe.

[7] Gonzales S.J., Reyes R.A., Braddom A.E., Batugedara G., Bol S., Bunnik E.M. (2020). Naturally Acquired Humoral Immunity Against Plasmodium Falciparum Malaria. Front. Immunol..

[8] Noor A.M., Kirui V.C., Brooker S.J., Snow R.W. (2009). The Use of Insecticide Treated Nets by Age: Implications for Universal Coverage in Africa. BMC Public Health.

[9] Alves F.P., Gil L.H.S., Marrelli M.T., Ribolla P.E.M., Camargo E.P., Da Silva L.H.P. (2005). Asymptomatic Carriers of *Plasmodium* spp. as Infection Source for Malaria Vector Mosquitoes in the Brazilian Amazon. J. Med. Entomol..

[10] Zaw M.T., Thant M., Hlaing T.M., Aung N.Z., Thu M., Phumchuea K., Phusri K., Saeseu T., Yorsaeng R., Nguitragool W. (2017). Asymptomatic and Sub-Microscopic Malaria Infection in Kayah State, Eastern Myanmar. Malar. J..

[11] Zoghi S., Mehrizi A.A., Raeisi A., Haghdoost A.A., Turki H., Safari R., Kahanali A.A., Zakeri S. (2012). Survey for Asymptomatic Malaria Cases in Low Transmission Settings of Iran under Elimination Programme. Malar. J..

[12] Shekalaghe S.A., Bousema J.T., Kunei K.K., Lushino P., Masokoto A., Wolters L.R., Mwakalinga S., Mosha F.W., Sauerwein R.W., Drakeley C.J. (2007). Submicroscopic Plasmodium Falciparum Gametocyte Carriage Is Common in an Area of Low and Seasonal Transmission in Tanzania. Trop. Med. Int. Health.

[13] Zhou Z., Mitchell R.M., Kariuki S., Odero C., Otieno P., Otieno K., Onyona P., Were V., Wiegand R.E., Gimnig J.E. (2016). Assessment of Submicroscopic Infections and Gametocyte Carriage of Plasmodium Falciparum during Peak Malaria Transmission Season in a Community-Based Cross-Sectional Survey in Western Kenya, 2012. Malar. J..

[14] Schneider P., Bousema J.T., Gouagna L.C., Otieno S., van de Vegte-Bolmer M., Omar S.A., Sauerwein R.W. (2007). Submicroscopic Plasmodium Falciparum Gametocyte Densities Frequently Result in Mosquito Infection. Am. J. Trop. Med. Hyg..

[15] Masanja I.M., Selemani M., Amuri B., Kajungu D., Khatib R., Kachur S.P., Skarbinski J. (2012). Increased Use of Malaria Rapid Diagnostic Tests Improves Targeting of Anti-Malarial Treatment in Rural Tanzania: Implications for Nationwide Rollout of Malaria Rapid Diagnostic Tests. Malar. J..

[16] Albertini A., Lee E., Coulibaly S.O., Sleshi M., Faye B., Mationg M.L., Ouedraogo K., Tsadik A.G., Feleke S.M., Diallo I. (2012). Malaria Rapid Diagnostic Test Transport and Storage Conditions in Burkina Faso, Senegal, Ethiopia and the Philippines. Malar. J..

[17] WHO (2018). Malaria Rapid Diagnostic Test Performance. Results of WHO Product Testing of Malaria RDTs: Round 8.

[18] Milne L.M., Kyi M.S., Chiodini P.L., Warhurst D.C. (1994). Accuracy of Routine Laboratory Diagnosis of Malaria in the United Kingdom. J. Clin. Pathol..

[19] Li P., Zhao Z., Wang Y., Xing H., Parker D.M., Yang Z., Baum E., Li W., Sattabongkot J., Sirichaisinthop J. (2014). Nested PCR Detection of Malaria Directly Using Blood Filter Paper Samples from Epidemiological Surveys. Malar. J..

[20] Kaaya R.D., Kajeguka D.C., Matowo J.J., Ndaro A.J., Mosha F.W., Chilongola J.O., Kavishe R.A. (2021). Predictive Markers of Transmission in Areas with Different Malaria Endemicity in North-Eastern Tanzania Based on Seroprevalence of Antibodies against Plasmodium Falciparum. BMC Res. Notes.

[21] Kassam N.A., Kaaya R.D., Damian D.J., Schmiegelow C., Kavishe R.A., Alifrangis M., Wang C.W. (2021). Ten Years of Monitoring Malaria Trend and Factors Associated with Malaria Test Positivity Rates in Lower Moshi. Malar. J..

[22] World Health Organization (2010). Basic Malaria Microscopy 2010.

[23] Miguel R.B., Coura J.R., Samudio F., Suárez-Mutis M.C. (2013). Evaluation of Three Different DNA Extraction Methods from Blood Samples Collected in Dried Filter Paper in Plasmodium Subpatent Infections from the Amazon Region in Brazil. Rev. Inst. Med. Trop. Sao Paulo.

[24] Snounou G., Singh B. (2002). Nested PCR Analysis of Plasmodium Parasites. Methods Mol. Med..

[25] Altman D.G., Bland J.M. (1994). Diagnostic Tests 2: Predictive Values. BMJ.

[26] Emidi B., Kisinza W.N., Kaaya R.D., Malima R., Mosha F.W. (2017). Insecticide Susceptibility Status of Human Biting Mosquitoes in Muheza, Tanzania. Tanzan. J. Health Res..

[27] Kaindoa E.W., Matowo N.S., Ngowo H.S., Mkandawile G., Mmbando A., Finda M., Okumu F.O. (2017). Interventions That Effectively Target Anopheles Funestus Mosquitoes Could Significantly Improve Control of Persistent Malaria Transmission in South-Eastern Tanzania. PLoS ONE.

[28] Kitau J., Oxborough R.M., Tungu P.K., Matowo J., Malima R.C., Magesa S.M., Bruce J., Mosha F.W., Rowland M.W. (2012). Species Shifts in the Anopheles Gambiae Complex: Do LLINs Successfully Control Anopheles Arabiensis?. PLoS ONE.

[29] Hayuma P.M., Wang C.W., Liheluka E., Baraka V., Madebe R.A., Minja D.T.R., Misinzo G., Alifrangis M., Lusingu J.P.A. (2021). Prevalence of Asymptomatic Malaria, Submicroscopic Parasitaemia and Anaemia in Korogwe District, North-Eastern Tanzania. Malar. J..

[30] Dalrymple U., Arambepola R., Gething P.W., Cameron E. (2018). How Long Do Rapid Diagnostic Tests Remain Positive after Anti-Malarial Treatment?. Malar. J..

[31] Gatton M.L., Ciketic S., Barnwell J.W., Cheng Q., Chiodini P.L., Incardona S., Bell D., Cunningham J., González I.J. (2018). An Assessment of False Positive Rates for Malaria Rapid Diagnostic Tests Caused by Non-Plasmodium Infectious Agents and Immunological Factors. PLoS ONE.

[32] Iqbal J., Sher A., Rab A. (2000). Plasmodium Falciparum Histidine-Rich Protein 2-Based Immunocapture Diagnostic Assay for Malaria: Cross-Reactivity with Rheumatoid Factors. J. Clin. Microbiol..

[33] Watson O.J., Sumner K.M., Janko M., Goel V., Winskill P., Slater H.C., Ghani A., Meshnick S.R., Parr J.B. (2019). False-Negative Malaria Rapid Diagnostic Test Results and Their Impact on Community-Based Malaria Surveys in Sub-Saharan Africa. BMJ Glob. Health.

[34] Unwin V.T., Ahmed R., Noviyanti R., Puspitasari A.M., Utami R.A.S., Trianty L., Lukito T., Syafruddin D., Poespoprodjo J.R., Santana-Morales M.A. (2020). Use of a Highly-Sensitive Rapid Diagnostic Test to Screen for Malaria in Pregnancy in Indonesia. Malar. J..

[35] Anthony M. (2002). Rapid Diagnostic Tests for Malaria Parasites. Clin. Microbiol. Rev..

[36] Tadesse E., Workalemahu B., Shimelis T. (2016). Diagnostic performance evaluation of the sd bioline malaria antigen ag pf/pan test (05fk60) in a malaria endemic area of southern Ethiopia. Rev. Inst. Med. Trop. Sao Paulo.

[37] Tseroni M., Pervanidou D., Tserkezou P., Rachiotis G., Pinaka O., Baka A., Georgakopoulou T., Vakali A., Dionysopoulou M., Terzaki I. (2015). Field Application of SD Bioline Malaria Ag Pf/Pan Rapid Diagnostic Test for Malaria in Greece. PLoS ONE.

[38] Cohee L.M., Nankabirwa J.I., Greenwood B., Djimde A., Mathanga D.P. (2021). Time for Malaria Control in School-Age Children. Lancet Child Adolesc. Health.

[39] Olapeju B., Choiriyyah I., Lynch M., Acosta A., Blaufuss S., Filemyr E., Harig H., Monroe A., Selby R.A., Kilian A. (2018). Age and Gender Trends in Insecticide-Treated Net Use in Sub-Saharan Africa: A Multi-Country Analysis. Malar. J..

[40] Chacky F., Runge M., Rumisha S.F., Machafuko P., Chaki P., Massaga J.J., Mohamed A., Pothin E., Molteni F., Snow R.W. (2018). Nationwide School Malaria Parasitaemia Survey in Public Primary Schools, the United Republic of Tanzania. Malar. J..

[41] Cohee L.M., Opondo C., Clarke S.E., Halliday K.E., Cano J., Shipper A.G., Barger-Kamate B., Djimde A., Diarra S., Dokras A. (2020). Preventive Malaria Treatment among School-Aged Children in Sub-Saharan Africa: A Systematic Review and Meta-Analyses. Lancet Glob. Health.

[42] Mathanga D.P., Halliday K.E., Jawati M., Verney A., Bauleni A., Sande J., Ali D., Jones R., Witek-McManus S., Roschnik N. (2015). The High Burden of Malaria in Primary School Children in Southern Malawi. Am. Soc. Trop. Med. Hyg..

[43] Okell L.C., Bousema T., Griffin J.T., Ouédraogo A.L., Ghani A.C., Drakeley C.J. (2012). Factors Determining the Occurrence of Submicroscopic Malaria Infections and Their Relevance for Control. Nat. Commun..

[44] Walldorf J.A., Cohee L.M., Coalson J.E., Bauleni A., Nkanaunena K., Kapito-Tembo A., Seydel K.B., Ali D., Mathanga D., Taylor T.E. (2015). School-Age Children Are a Reservoir of Malaria Infection in Malawi. PLoS ONE.

[45] Yazdani S.S., Mukherjee P., Chauhan V.S., Chitnis C.E. (2006). Immune Responses to Asexual Blood-Stages of Malaria Parasites. Curr. Mol. Med..

[46] Boyd M.F., Stratman-Thomas W.K., Kitchen S.F. (1936). On Acquired Immunity to Plasmodium Falciparum1. Am. J. Trop. Med. Hyg..

[47] Cabrera E.J., Barr M.L., Silverman P.H. (1977). Long-Term Studies on Rhesus Monkeys (Macaca Mulatta) Immunized against Plasmodium Knowlesi. Infect. Immun..

[48] Wilson R.J., Phillips R.S. (1976). Method to Test Inhibitory Antibodies in Human Sera to Wild Populations of Plasmodium Falciparum. Nature.

[49] Griffin J.T., Déirdre Hollingsworth T., Reyburn H., Drakeley C.J., Riley E.M., Ghani A.C. (2015). Gradual Acquisition of Immunity to Severe Malaria with Increasing Exposure. Proc. R. Soc. B Biol. Sci..

[50] van den Hoogen L.L., Walk J., Oulton T., Reuling I.J., Reiling L., Beeson J.G., Coppel R.L., Singh S.K., Draper S.J., Bousema T. (2019). Antibody Responses to Antigenic Targets of Recent Exposure Are Associated With Low-Density Parasitemia in Controlled Human Plasmodium Falciparum Infections. Front. Microbiol..

[51] Mensah B.A., Myers-Hansen J.L., Obeng Amoako E., Opoku M., Abuaku B.K., Ghansah A. (2021). Prevalence and Risk Factors Associated with Asymptomatic Malaria among School Children: Repeated Cross-Sectional Surveys of School Children in Two Ecological Zones in Ghana. BMC Public Health.

[52] Worku L., Damtie D., Endris M., Getie S., Aemero M. (2014). Asymptomatic Malaria and Associated Risk Factors among School Children in Sanja Town, Northwest Ethiopia. Int. Sch. Res. Not..

[53] Berzosa P., de Lucio A., Romay-Barja M., Herrador Z., González V., García L., Fernández-Martínez A., Santana-Morales M., Ncogo P., Valladares B. (2018). Comparison of Three Diagnostic Methods (Microscopy, RDT, and PCR) for the Detection of Malaria Parasites in Representative Samples from Equatorial Guinea. Malar. J..

[54] Acquah F.K., Donu D., Obboh E.K., Bredu D., Mawuli B., Amponsah J.A., Quartey J., Amoah L.E. (2021). Diagnostic Performance of an Ultrasensitive HRP2-Based Malaria Rapid Diagnostic Test Kit Used in Surveys of Afebrile People Living in Southern Ghana. Malar. J..

[55] Poti K.E., Sullivan D.J., Dondorp A.M., Woodrow C.J. (2020). HRP2: Transforming Malaria Diagnosis, but with Caveats. Trends Parasitol..

[56] Oladosu O., Adedokun Victoria A., Adeniyi Akinkunle V., Oyibo Wellington A. (2021). Performance Evaluation of Malaria HRP-2 Rapid Diagnostic Test among Febrile Patients with Malaria in Iwo, Osun State, Nigeria. Int. J. Trop. Dis..

[57] Baum E., Sattabongkot J., Sirichaisinthop J., Kiattibutr K., Jain A., Taghavian O., Lee M.C., Huw Davies D., Cui L., Felgner P.L. (2016). Common Asymptomatic and Submicroscopic Malaria Infections in Western Thailand Revealed in Longitudinal Molecular and Serological Studies: A Challenge to Malaria Elimination. Malar. J..

[58] van Eijk A.M., Sutton P.L., Ramanathapuram L., Sullivan S.A., Kanagaraj D., Priya G.S.L., Ravishankaran S., Asokan A., Sangeetha V., Rao P.N. (2019). The Burden of Submicroscopic and Asymptomatic Malaria in India Revealed from Epidemiology Studies at Three Varied Transmission Sites in India. Sci. Rep..

[59] Santos L., Pereira N.R., Andrade P., Dias P.F., Alves C.L., Abreu C., Serrão R., Ribeiro M., Sarmento A. (2015). Prozone-like Phenomenon in Travellers with Fatal Malaria: Report of Two Cases. J. Infect. Dev. Ctries..

[60] Luchavez J., Baker J., Alcantara S., Belizario V., Cheng Q., McCarthy J.S., Bell D. (2011). Laboratory Demonstration of a Prozone-like Effect in HRP2-Detecting Malaria Rapid Diagnostic Tests: Implications for Clinical Management. Malar. J..

[61] Sutanto I., Kosasih A., Elyazar I.R.F., Simanjuntak D.R., Larasati T.A., Dahlan M.S., Wahid I., Mueller I., Koepfli C., Kusriastuti R. (2018). Negligible Impact of Mass Screening and Treatment on Mesoendemic Malaria Transmission at West Timor in Eastern Indonesia: A Cluster-Randomized Trial. Clin. Infect. Dis..

[62] Cook J., Xu W., Msellem M., Vonk M., Bergström B., Gosling R., Al-Mafazy A.-W., McElroy P., Molteni F., Abass A.K. (2015). Mass Screening and Treatment on the Basis of Results of a Plasmodium Falciparum-Specific Rapid Diagnostic Test Did Not Reduce Malaria Incidence in Zanzibar. J. Infect. Dis..

